# Supplementary material for: OsSHMT4 Is Required for Synthesis of Rice Storage Protein and Storage Organelle Formation in Endosperm Cells
Source: Plants (Basel). 2023 Dec 26;13(1):81. doi: 10.3390/plants13010081 (PMC10780996; doi:10.3390/plants13010081)
Supplement: Supplementary file 1 [file plants-13-00081-s001.zip › Supplemental Figures.pdf]

Supplemental Figures for

OsSHMT4 Is Required for Synthesis of Rice Storage Protein and Storage Organelle Formation in Endosperm Cells

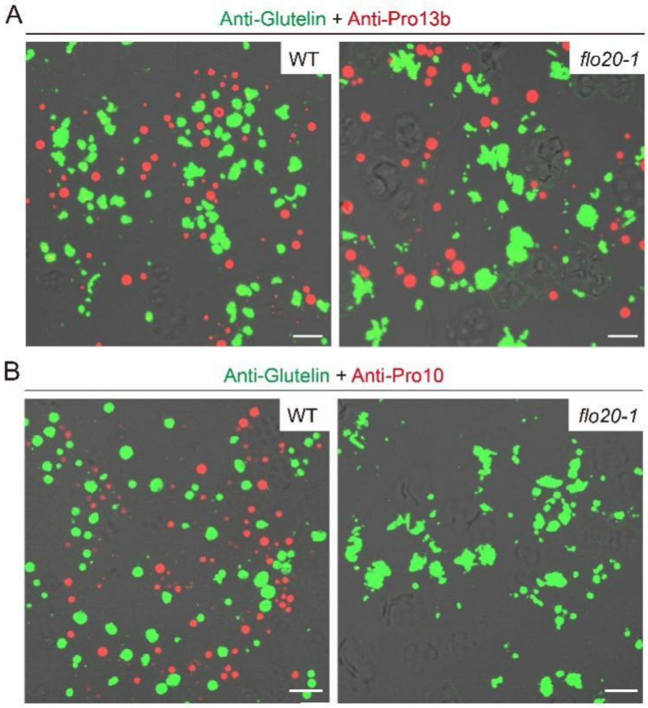

Supplemental Figure 1. Double immunofluorescence microscopy of glutelin and prolamins in the developing subaleurone cells of the WT and *flo20-1* endosperm at 9 DAF. Secondary antibodies labeled with Alexa Fluor 488 (green) and Alexa Fluor 555 (red) were used to detect antigens recognized by the monoclonal anti-glutelin antibodies from mice and polyclonal anti- Pro 13b (A) or anti- Pro 10 (B) antibodies from rabbits, respectively. Bars = 5 µm.
